# Analytical Validation and Assessment of Baseline Fecal Glucocorticoid Metabolites in Northern Sea Otters (*Enhydra lutris kenyoni*) in Human Care

**DOI:** 10.3390/ani13132175

**Published:** 2023-07-02

**Authors:** Amy Y. Olsen, Angela Smith, Caroline Hempstead, Shawn E. Larson

**Affiliations:** Seattle Aquarium, 1483 Alaskan Way, Seattle, WA 98101, USA; a.smith@seattleaquarium.org (A.S.); c.hempstead@seattleaquarium.org (C.H.); s.larson@seattleaquarium.org (S.E.L.)

**Keywords:** hormones, stress, marine mammal, endocrinology, cortisol, corticosterone, enzyme immunoassay, fecal glucocorticoid metabolites

## Abstract

**Simple Summary:**

Animals in human care, such as northern sea otters (*Enhydra lutris kenyoni*), allow for long-term studies of the stress response to environmental stimuli, husbandry, and medical procedures. The goal of this study was to analytically validate the use of two commercially available enzyme immunoassay kits to measure fecal glucocorticoid metabolites (FGMs), specifically cortisol-immunoreactive and corticosterone-immunoreactive metabolites. Individual baseline levels of four adult sea otters were calculated, each of which were found to be unique. We attempted to match FGM peaks with caregiver notes on environmental enrichment, husbandry changes, and medical procedures. This work provides insight into effective husbandry management to improve the welfare of sea otters and other marine mammals under human care.

**Abstract:**

Fecal glucocorticoid metabolites (FGMs) have been used as a non-invasive and indirect measurement of the complex stress response in a variety of species. Animals in facilities under managed care allow for the longitudinal study of FGMs in a controlled environment. Animal histories often include environmental, husbandry, and medical notes that can be matched to FGM concentrations to aid in the physiological validation of adrenal stimulation and response. The goal of this study was to demonstrate analytical validations using two enzyme-linked immunosorbent assays (EIA) to measure FGMs from northern sea otters (*Enhydra lutris kenyoni*) under human care (Seattle Aquarium, Seattle, WA, USA) and to determine baseline and stress response spike levels for individual sea otters. Individual variation was found among the four subjects in the study with fecal baseline levels ranging from 20.2 to 83.7 ng/g for cortisol-immunoreactive metabolites and 52.3 to 102 ng/g for corticosterone-immunoreactive metabolites. As a retrospective study, 39 percent of hormone peaks were associated with notes and most FGM spikes were associated with veterinary procedures or days in which enrichment items were provided and produced an excitatory response. Monitoring baseline FGMs levels and events associated with hormone peak values may provide insight into effective husbandry management to improve the overall welfare of sea otters and other marine mammals.

## 1. Introduction

Sea otters (*Enhydra lutris*) are popular exhibit animals in zoos and aquariums. These animals under human care provide an avenue for visitors to learn about biology and ecology, and to view an animal they might never have the opportunity to see in the wild. Both northern (*E.l. kenyoni*) and southern *(E.l. nereis*) subspecies are displayed in facilities throughout the United States [1]. The United States Fish and Wildlife Service (USFWS) is responsible for their conservation and management, and issues Letters of Authorization for their display within facilities. These facilities must follow guidelines set by the Animal Welfare Act and enforced by the United States Department of Agriculture Animal and Plant Health Inspection Service (USDA APHIS). The vast majority of sea otter facilities in North America are also accredited by the Association of Zoos and Aquariums (AZA). Accreditation involves a lengthy process that includes inspections and considerations such as minimum husbandry criteria (enclosure size, diet, number of animals kept together, etc.), number of trained staff, animal welfare, water quality, cleanliness of facility, and many others [2]. As of 2022, there were 15 facilities in North America displaying 61 sea otters from both the southern and northern subspecies [1]. 

North American AZA-accredited zoos and aquariums strive to ensure the well-being of animals in their care, by limiting or managing the relative “stress” levels of these animals, a priority in many animal welfare programs. Stress is typically defined as an individual’s perception of and response to an external threat to survival. Energy investments in processes such as reproduction, growth, and the immune system are reduced to focus energy on the immediate threat [3]. Stress responses can differ in length of time and intensity, with different repercussions on health. Acute stress is defined as being short in duration or a spike that can boost energy, in which an animal may be able to defend itself, adapt, or cope with the stimulus with little detrimental long-term effects. Chronic stress is longer in duration and repeated, leading to an inability to adapt to changing conditions and, consequently, to health problems such as decreased immune response and reproduction in many wildlife species [4].

When a vertebrate (and some invertebrate [5]) animal perceives a stressor, the sympathetic nervous system activates the flight-or-fight response and initiates the hypothalamic–pituitary–adrenal (HPA) axis [6,7,8]. This neuroendocrine system involves a cascade of hormones, which can lead to the synthesis and release of the glucocorticoid hormones (GCs), cortisol and corticosterone, from the adrenal cortex. This system regulates blood circulation, digestion, the immune system, reproduction, and energy storage and expenditure all to mobilize energy and mental readiness for the potential “fight or flight” to deal with or remove the animal from the stressor [6]. Cortisol is considered the primary GC stress response hormone in fish and most mammals [9]. Corticosterone is considered the primary GC stress response hormone in amphibians, reptiles, some rodents, and birds [9].

Circulating GC levels vary within individuals throughout the day and fluctuate based on circadian rhythms, time of day, season, sex, age, social status, reproductive phase, immune health, and growth [3,10,11]. Glucocorticoid hormones are metabolized from the blood and excreted in bodily fluids including urine and feces, referred to as fecal glucocorticoid metabolites (FGMs) [12,13]. These metabolites are highly transformed byproducts and contain very little parent compound [14]. Fecal samples are thought to represent the circulating metabolized GCs experienced by the animal over a longer period of time rather than a single serum sample due to pooling effect of metabolites over a longer period [4,15].

Methods describing the long-term measurement of fecal reproductive hormone metabolites (total estrogens and progestogens) from sea otter feces have been validated using radioimmunoassay methods (RIA) [16] and enzyme-linked immunosorbent assays (EIA) [17]. Enzyme-linked immunosorbent assays have become a popular method for many laboratories because they do not require the use of hazardous radioactive reagents (RIA) and are relatively inexpensive compared to RIA or mass spectrometry.

In a study on one captive northern sea otter, both FGMs were found to be stimulated by the ACTH challenge but cortisol was more elevated than corticosterone [18]. In wild northern sea otter serum collections, circulating corticosterone was thought to be the active GC, as it is more significantly related to capture stress than cortisol [19]. However, new research has demonstrated that cortisol is the dominant circulating serum GC in captive southern sea otters [20]. Due to the competitive nature of the immunoassay method, cross-reactions must be considered [14], and this study analyzed both to be conservative.

Animals under managed human care provide a unique opportunity to study individual stress response to a variety of variables over time. These animals are housed in a controlled environment in which high-quality food is offered and external stressors such as predation or extreme environmental conditions are either minimized or non-existent. By removing these stressors and collecting samples over years across seasons in a non-invasive way (i.e., opportunistically collected fecal samples), baseline FGMs can be established for individuals [21]. These samples can also be used for long-term monitoring and can be linked to behavioral, environmental, and health documentation to identify perturbation or events that may have caused acute or chronic spikes relative to baseline values [21,22,23]. To date, there have been no longitudinal studies reporting FGM baseline levels in sea otters.

In this study, animals were not exposed to threats to survival such as predators or extreme environmental conditions, but stimuli that were deemed potential stressors included natural animal interactions (mating, reproduction, or playing), environmental variables (construction activity or noise), health related issues (lethargy or low interest, surgery, etc.), and providing certain environmental enrichment items. A unique stimulus specific to animals in human care is the addition of items provided as environmental enrichment. This is a common animal husbandry principle that provides an opportunity for an animal to optimize their level of mental stimulation and satisfy their behavioral needs, and to create a variable environment [24]. Items were chosen to encourage certain behaviors and social interactions or for tactile stimulation and, at the SA, must be approved by the lead mammal keeper and veterinarian. Examples include imitation kelp strips, frozen ice treats, mirrors, plastic Boomer Balls^®^, and various feeder puzzles.

The goal of this study was to validate the use of commercially available EIA kits to measure FGMs in archived northern sea otter fecal samples and to determine baseline and stress-response FGM levels for individual sea otters of different sex and age classes. An additional goal was to link FGM stress response peaks to animal husbandry notes and histories to determine potential stressors for individual animals in managed care. Establishing the individual FGM baseline and stress response may provide insight into improving husbandry methods and increasing the welfare of these endangered animals.

## 2. Materials and Methods

### 2.1. Sea Otter Subjects: Exhibit Description and Husbandry

The northern sea otter subjects in this study were housed at the Seattle Aquarium (SA), an AZA-accredited facility. All individuals were maintained on public display in a mixed sex group (3 females and 1 male). Three of the animals (F1, F3, and M1) were born in the wild and were subsequently rescued, rehabilitated as pups, and transferred to SA for long-term care. One female, F2, was born at the SA to F1. As of 2016, at the end of the study period, the ages of the animals were 2 (F3), 14 (F2), 17 (M1), and 19 (F1) years old. Sea otters reach reproductive maturity between ages 2 and 5 [25]. The sea otter diet generally comprised of crustaceans, mollusks, and fish [26]. Food was offered eight times a day from 07:00 to 21:00 h. Average daily food intake ranged from 4.5 to 5.7 kg per otter depending on individual sea otter weights, equivalent to 16–20% of their body weight [27]. The sea otter enclosure has three pools that vary in depth between 1 and 4 m and water volumes ranging between 71,355 and 203,882 L. Animals were held in natural seawater (average salinity 28 ppt and temperature 10–12 °C) drawn in from Puget Sound and sand filtered before use [28]. Water quality was monitored in accordance with United States Department of Agriculture (USDA) and Washington State Department of Ecology requirements. All animals received behavioral conditioning (training) to allow effective moving of animals between exhibits, general husbandry procedures, veterinary care, and enrichment. 

### 2.2. Sample Collection and Processing

Fecal samples were collected opportunistically during or immediately after sea otter feedings between 1997 and 2016 during daylight hours of 07:00 to 16:00. Individual otter samples were collected over a period ranging from 2 to 17 years (Table 1). Samples were collected from the water immediately after the animal voided using a fine mesh net, then transferred to labeled 100 mL plastic specimen containers. Samples were refrigerated between 0–4 °C (<24 h) or frozen immediately (−80 °C) until further processing. At the SA in recent years, there has been increased effort for biologists to collect husbandry and behavioral notes as often as possible to describe significant observations during the time a fecal sample was collected. Such notes included animal behavioral activity such as mating, increased or decreased activity levels (lethargy and aggression), as well as outside influences such as construction and city noise (helicopters, boats, and fireworks).

Fecal GC metabolites were extracted by a previously described technique [17]. Briefly, samples were oven-dried at 60 °C overnight or until completely dried. Fecal metabolites may not be evenly distributed within the sample so dried samples were pulverized and mixed [13,29]. Following homogenization, 0.20 ± 0.02 g subsamples were extracted with 2 mL of 90% methanol (Carolina Biological, Burlington, NC, USA). Fecal samples that were below this target weight were discarded from the analysis and not recorded. Capped sample vials were placed on a plate shaker for 30 min, then centrifuged at high speed for 30 min. The remaining supernatant was pipetted into a clean microcentrifuge tube. Sample extractions were stored frozen at −80 °C until hormone analysis. All fecal samples were dried within one week and extracted within one month, and extractions were assayed within four months of collection. Assays were run over the entire study period. 

### 2.3. Assay Methods—Enzyme-Linked Immunosorbent Assay (EIA)

Sample extractions were run on EIAs for cortisol (#ADI-900-071) and corticosterone (#ADI-900-097) using commercially available kits per manufacturer’s specifications (Enzo Life Sciences Inc., New York, NY, USA). All assay plates included a full standard curve, and duplicate non-specific binding (NSB) wells, total activity (TA) wells, zero standard (Blank) wells, and maximum binding (B0) wells. Briefly, all reagents were brought to room temperature before use. The following reagents were then pipetted into appropriate wells on the assay plate: 100 µL assay buffer into B0 wells, 150 µL into NSB wells, 100 µL of standards (#1–#7 for cortisol and #1–#5 for corticosterone), 100 µL of sample dilutions (described below), 50 µL of blue conjugate (except in TA and blank wells), and 50 µL of yellow antibody (except in blank, TA, and NSB wells). For cortisol kits, blue conjugate was a solution of alkaline phosphatase conjugated with cortisol, and yellow antibody was a solution of a mouse monoclonal antibody to cortisol. For corticosterone kits, blue conjugate was a solution of alkaline phosphatase conjugated with corticosterone, and yellow antibody was a solution of a sheep polyclonal antibody to corticosterone. Plates were then covered with plate sealer and incubated at room temperature on a plate shaker for 2 h at ~500 rpm. Plates were then washed with 400 µL of wash solution three times, 5 µL of blue conjugate was added to TA wells, and 200 µL of pNpp substrate solution (p-nitrophenyl phosphate in buffer) was added to all wells. The plates were then incubated at room temperature in the dark for 1 h without shaking. Then, 50 µL of stop solution (trisodium phosphate in water) was added to every well before being read on plate reader. Assay standard range, sensitivity, and cross-reactivities are reported in kit inserts (see Supplementary Table S1). All samples, standards, and controls were assayed in duplicate, with the resulting coefficient of variation (CV) between all duplicates required to be <10% for acceptance. A sample with CV higher than 10% was re-run on another assay plate. If a set of standards resulted in high CVs, the entire plate was run again. If more than 50% of samples on a plate had high CVs, the entire plate was run again. Acceptable inter-assay CVs were required to be <16% and intra-assay CVs were required to be <10%. Plates were read on a ChroMate^®^ plate reader (Awareness Technologies, Westport, CT, USA) at optical density of 405 nm. ChroMate^®^ Manager software (ver 6.3.1.244) was used to calculate concentration by fitting a 4-parameter logistic curve. 

Parallelism and accuracy tests were run for each FGM for analytical assay validation. Parallelism was used to determine whether the sample is immunologically similar to the standard and can be measured proportionately (i.e., is the assay measuring what it should be measuring?). Parallelism was tested by running a serially diluted pool of four samples alongside hormone standards. Dilutions included neat (no dilution), 1:2, 1:4, 1:8, 1:16, 1:32, and 1:64 with assay buffer provided in the kit. Sample dilution rates are based on concentrations of pooled samples that result in 50% assay antibody binding, providing the most accurate value in the middle of the standard curve. The parallelism graph for cortisol-immunoreactive metabolites revealed 50% binding of pooled sample at a 1:6 dilution; however, a minimum of 1:10 dilution is recommended to avoid matrix interference of the methanol solvent [30]. Therefore, all samples were run at a 1:10 dilution. The parallelism graph for corticosterone-immunoreactive metabolites revealed a 50% binding at a 1:30 dilution, and samples for this assay were run at this dilution. Both cortisol-immunoreactive and corticosterone-immunoreactive metabolites were present in detectable concentrations on the standard curve. Accuracy was tested by spiking a set of hormone standards with a pool of seven fecal extracts to see if this interfered with standard sample assay binding. The same pool of samples was used for each hormone assay. 

Samples were diluted with assay buffer, and most samples had hormone levels that fell within the standard curve. Samples above the maximum detection limit of the assay were diluted further and run-on subsequent assays. Values that were below the minimum detection limit of the assay were assigned a value halfway between zero and the minimum standard of the assay for statistical analysis purposes (0.78 ng/g for the cortisol assay and 0.16 ng/g for the corticosterone assay). However, it is understood that the actual absolute value is somewhere between zero and the minimum of the assay. Final concentrations were expressed as ng/g of dry fecal weight.

### 2.4. Baseline and Peak Calculations

Mean values per individual were calculated first by averaging all data points across time, then values one standard deviation (SD) above the mean were removed and mean was recalculated to determine baseline for each hormone and animal (modified from [31,32,33,34]). 

Acute stress response was defined here as a single sample in which both FGMs had peaks above baseline or >2SD above the mean. Chronic stress was defined here as multiple days or weeks of samples in which both FGM metabolites were >2SD above the mean. Only data points in which both hormone metabolites peaked were considered as an adrenal stress response, due to cross-reactivity in the immunoassays [14].

### 2.5. Statistical Analysis

Statistics were calculated in R (version 4.0.3; [35]) using the packages tidyverse [36], here [37], ggbreak [38], and cowplot [39]. Parallelism results were transformed and graphed as log_10_ of standard concentration to percent antibody bound. Values were assessed with an ANCOVA F test (α = 0.05) to determine variance of slope for each hormone metabolite. Accuracy results were graphed by comparing expected standard concentration to observed standard concentrations. Final results were analyzed with linear regression, with acceptable limits of r^2^ > 0.95 and slope between 0.8–1.2 for acceptance. 

## 3. Results

### 3.1. Analytical Validation—Parallelism and Accuracy

**Figure 1:**
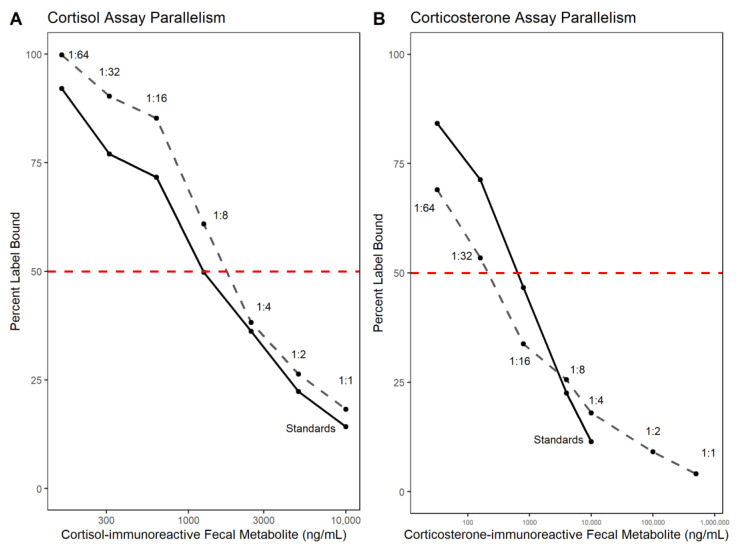
Parallelism graphs for cortisol-immunoreactive and corticosterone-immunoreactive fecal metabolites in sea otters for enzyme immunoassay validation. Serially diluted standards (solid line) are graphed with diluted extracts (dashed line). The horizontal lines (red) indicate the 50% binding point. Both cortisol and corticosterone assays showed parallelism to the standard curve, indicating the sample is immunologically similar to the standard and can be measured proportionately. (**A**) Cortisol Assay Parallelism. (**B**) Corticosterone Assay Parallelism.

**Figure 2:**
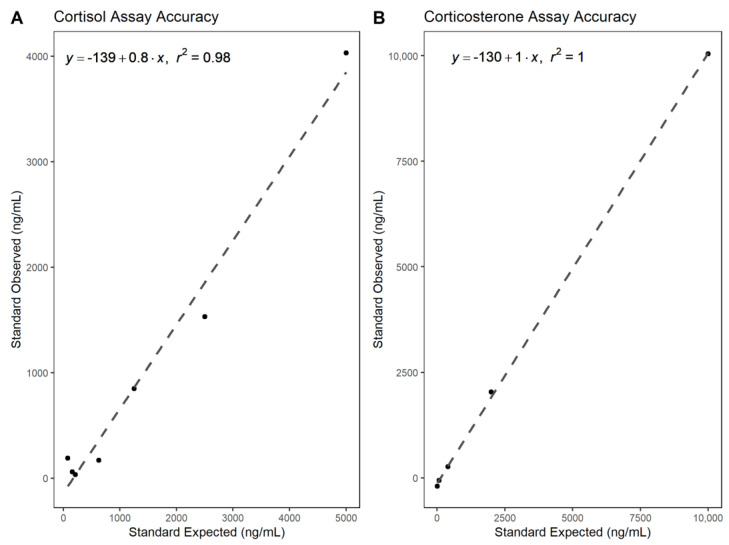
Accuracy graphs for fecal cortisol-immunoreactive and corticosterone-immunoreactive metabolites in sea otters for enzyme immunoassay validation. Accuracy was tested by spiking a set of hormone standards with a pooled sample mix to check if the measured concentration matches the true concentration by subtracting the known addition. This tests for any interference within the sample matrix. It was determined that the hormones in the samples or other metabolites had not interfered with assay antibody binding. However, note that low concentrations may be inaccurate, due to the clustering observed at the low end of each axes. (**A**) Cortisol Assay Accuracy. (**B**) Corticosterone Assay Accuracy.

Enzyme immunoassay validation was achieved by demonstrating parallelism among absorbance graphs of serial diluted standards and pooled samples (N = 4; three female and one male). Both cortisol and corticosterone assays showed parallelism to the standard curve (Figure 1), with the cortisol assay producing a more parallel result to the standard curve. Cortisol assay parallelism F-test results were F_6_ = 1.254 and P = 0.395. Corticosterone assay parallelism F-test results were F_4_ = 2.193 and P = 0.232. Cortisol and corticosterone assay accuracy curves indicated the measured standard concentrations corresponded in a 1:1 fashion with the spiked standard curve upon subtracting the known concentration (r^2^ = 0.98 and r^2^ = 1.0, respectively; Figure 2). However, low concentrations may not be accurate as observed in the cluster of points at the low end of the accuracy graphs (Figure 2). Both accuracy tests were run at a 1:10 dilution of pooled samples. Cortisol assay inter- and intra- assay coefficients of variation were 15.6 and 10.3, respectively. Corticosterone assay inter-and intra- assay coefficients of variation were 12.7 and 13.6, respectively. 

### 3.2. Individual Longitudinal Data

**Figure 3:**
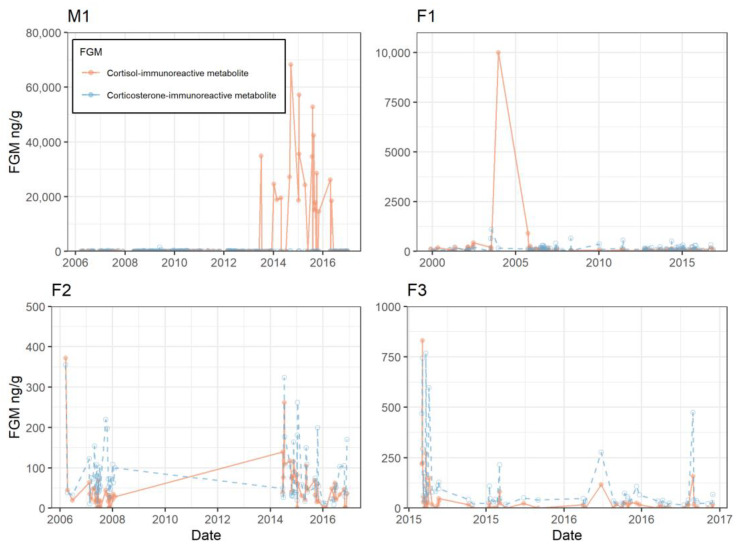
Profiles for four northern sea otter subjects in the study. M1 is a male, and F1, F2, and F3 are females. Fecal cortisol-immunoreactive metabolite (ng/g dry-weight feces) is shown (solid orange circle and line), along with fecal corticosterone-immunoreactive metabolite (ng/g dry-weight feces; open blue circle and dashed line). Note the variation in y-axis range and values.

**Figure 4:**
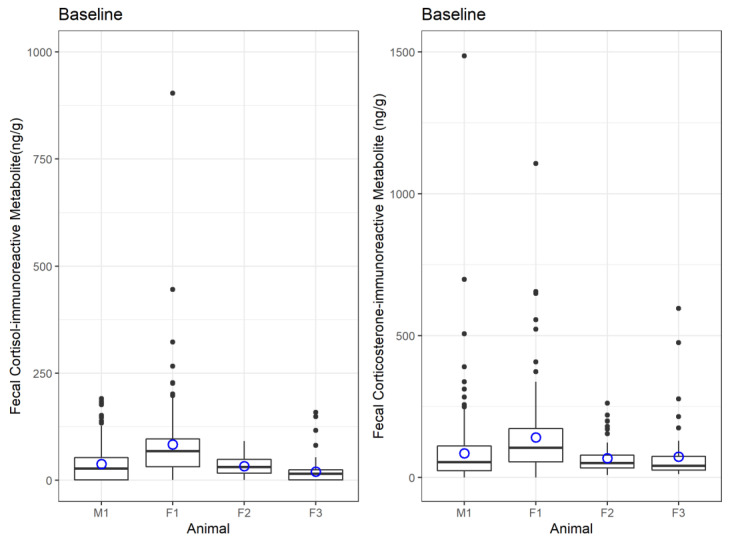
Boxplots of fecal cortisol-immunoreactive and corticosterone-immunoreactive metabolites (ng/g dry-weight feces) in four northern sea otter individuals (M1 is a male, and F1, F2, and F3 are females). Samples above one standard deviation were removed, and remaining samples were plotted (boxes show second and third quartile and black dots are above the third quartile). The mean of remaining values (baseline) is shown (blue dot). Overall, baseline cortisol-immunoreactive metabolite values were below 83.7 ng/g dry weight, and baseline corticosterone-immunoreactive metabolite values were below 102 ng/g dry weight.

Longitudinal profiles for all four subjects showed variable patterns and spikes throughout the study period (Figure 3). Individual sea otter overall mean and baseline values varied between animals and sex (Figure 4). Baseline cortisol-immunoreactive metabolite levels ranged from 20.2–83.7 ng/g dry-weight feces. Average cortisol-immunoreactive metabolite range for all four animals was 44.6–2525 ng/g dry weight, with peaks up to 68,233 ng/g (M1). Otter M1 had the highest overall cortisol-immunoreactive metabolite average and highest peaks; however, his baseline level was below the baseline of F1. Otter F1 (the oldest female) had the highest cortisol-immunoreactive metabolite baseline, and F3 (the youngest female) had the lowest baseline. Baseline corticosterone-immunoreactive metabolite levels ranged from 52.3 to 102 ng/g dry weight. Average corticosterone-immunoreactive metabolite averages ranged from 81.9 to 141 ng/g dry weight with peaks up to 1486 ng/g dry weight (M1). Otter F1 had the highest average and baseline level. 

Significant peaks, defined as those occurring >2SD above mean, were identified for each individual. Dates of peaks were matched against the biologists’ reports and notes the day before and day of the FGM peak (i.e., previous 48 h) to determine potential stress stimuli events. Health records, environmental notebooks, and enrichment records were evaluated. The number of peaks in each FGM, along with peaks in both, are presented (Table 2). Otter F3 had the highest number of peaks associated with notes (health related, normal behavior, exhibit shifts, and enrichment provided). 

## 4. Discussion

This study found that FGMs were detectable in sea otter fecal samples using these commercially available EIA kits. Analytical parallelism and accuracy validations were acceptable for both cortisol and corticosterone assays, although biological/physiological validations are still needed.

All sea otter subjects exhibited what we considered to be normal stress activity. There was variation between individual sea otter FGM baselines, which may be expected due to differences in temperament (i.e., a naturally cautious or nervous animal vs. a calm one), individual perception of external stimuli, previous experience, age, and sex [7]. Kolbe et al. [40] found that age had a significant impact on the level and circadian rhythm of FGMs in laboratory mice. The oldest animal in this study, F1, a 19-year-old female, had the highest baseline level for both fecal cortisol-immunoreactive and corticosterone-immunoreactive metabolites, while the only male otter, 17 years old, demonstrated the highest cortisol-immunoreactive peaks, most notably in the later years of the study as he aged between 2013–2016. The male otter did receive deslorelin implants in 2012, associated with a peak in both FGMs. Deslorelin, a gonadotropin-releasing hormone (GnRH) agonist, has been shown to decrease FGMs in clouded leopards [41], which may have been the case here for the period of effectiveness after implantation. The FGM peak associated with this note may have been a response to the vet exam and implantation process itself. In the time after the deslorelin effects wore off, the male fecal cortisol-immunoreactive metabolite values increased dramatically. Both older animals in this study (M1 and F1) exhibited much higher peaks than the younger subjects (F2 and F3), although we acknowledge they were monitored for a longer period of time and the high peaks occurred later in life.

Male otters are known to establish breeding territories [27,42] and this male (housed with a group of females) may have been acting as a territorial male which could explain his higher FGM values [11,43,44]. This male did not separate easily from the females (which was carried out when two of the females had pups), and he was also moved to another institution for four months. Upon his return, it was even more difficult to separate him from the females. In addition, he has been described as having a nervous temperament by his caregivers which also may explain his endocrine profile. Unlike the other otters housed at the SA, M1 had a noticeable reaction to external stimuli such as construction activity, helicopters, and boats traveling close to the aquarium, and had been observed performing repetitive overgrooming behaviors in response to these stimuli. Animals with “bold” personalities have been found to have lower glucocorticoid responses than “nervous/shy” individuals [13].

A study that measured glucocorticoid serum values within northern sea otters, *E.l. kenyoni*, reported cortisol values of 28.25 ng/mL for males and 58.45 ng/mL for females, and corticosterone values of 9.65 ng/mL for males and 15.05 ng/mL for females post capture using RIA techniques [31]. Chinn et al. [45] reported cortisol serum values of 94.97 ± 4.21 ng/mL from female northern sea otters using RIA and corticosterone blood values of 10.11 ± 0.61 ng/mL using enzyme immunoassay post capture. These studies were conducted on wild sea otter serum values which provides a shorter snapshot of the endocrine profile than fecal samples. Both of these RIA studies measured higher cortisol values than corticosterone. This EIA study found the opposite in fecal metabolite values, which may be due to the corticosterone assay picking up cortisol-immunoreactive metabolites (i.e., cross-reactivity). While it is not possible to compare the serum to fecal samples, nor concentrations across studies, further research should investigate the suite of corticosterone metabolites the EIA antibodies are binding to. Additional studies could compare EIAs from various manufacturers to compare results. 

Sea otter gut transit time is thought to be relatively short, between three to six hours, with 20 fecal samples collected from one male in 72 h post ACTH challenge and a peak of FGMs at six hours [18,34,42]. However, it is unknown how long the excitatory phase may last after natural stimulation from an outside source that is not an ACTH challenge; thus, the previous 48 h were used as the time frame in which to look for medical records and archived behavioral notes or histories. For example, if a stimulus (e.g., loud construction noise) was noted in the husbandry log, then peaks in hormones were evaluated only if they occurred within this time frame. This is an area in which more research should be carried out to see how long external stimuli (that is not a capture and an administered drug such as ACTH) result in elevated FGMs.

One limitation to this study was that samples were only collected during working hours, and it is known that most animals have circadian rhythms of GCs [46] which would not be reflected here. Samples were collected opportunistically by animal keepers and rarely were multiple samples collected in one day or even subsequent days from the same individual. Another limitation was that notes were sparse in the early years of fecal collection, and only within the last 10 years has record keeping improved dramatically. Animal keepers did not originally note when enrichment was provided, which likely occurred every day. Additionally, notes may have missed the “real” reason for peaks (e.g., a visitor making very loud noises). We did observe peaks that were associated with “normal behavior” notes (although “normal behavior” was not recorded until recent years). Sample size is another consideration, as we show that four individual sea otters vary in FGM baseline levels. A larger sample size may provide a wider view of how individual baselines compare in a population.

The advantage of studying animals under human care is access to historic health and behavioral records. However, because this study was a retrospective look at FGMs, there were many FGM spikes in which there were no associated significant notes on individual animal behavior, environment, or health. Historically, record keeping was sparse and only carried when a big event happened, but, in recent years, records have become more detailed and were taken multiple times a day. Out of 487 total samples analyzed, only 23 samples had peaks in both FGM assays, and, of these, only 9 samples had associated notes. Notes associated with spikes included deslorelin implants (it is unknown whether this was due to the procedure or the implants themselves), vet exams, arrival to SA, normal behavior, shifting exhibits, and providing certain environmental enrichment items. The most common associated note with peaks was health-related such as post veterinary procedure or recuperating from a surgery. It should be stated that when an animal under human care is sick or not behaving normally, there are often more observations made and more samples collected for health documentation. Thus, there may be a bias towards samples and notes taken when animals may be “stressed” with fewer husbandry notes and samples taken during times when the animal seems normal or non-stressed. More data are needed to investigate whether health procedures are followed by no peaks.

In addition to health-related metrics, one of the other activities associated with a spike in FGMs was the addition of enrichment items to the exhibit. Environmental enrichment is used to enhance the quality of life of an animal in human care by providing the behavioral stimuli for optimal physiological and psychological well-being. Husbandry training, modifying social groupings, introducing novel and sensory items, providing food in a variety of ways (e.g., within ice treats or feeder puzzles), or the use of permanent exhibit items may help stimulate natural behaviors and enhance animal welfare. Ice treats are a common form of environmental enrichment for sea otters living in a zoological setting. These stimuli could cause an excitatory response and a release of the adrenal FGMs resulting in a spike as reported here. Sea otters tend to play vigorously with enrichment items, causing energy expenditure and an excitatory response. There were notes on days in which enrichment items were provided and peaks did not occur. The peaks that were associated with enrichment activities were lower than peaks associated with health issues, suggesting the peaks may not be deleterious. Even chronically increased FGM levels during predictable life-history events are not necessarily deleterious [47]. Other biochemical stimuli (such as raising blood glucose) are known to also stimulate GC release [8,9,10] which could be the case with food hidden in ice treats.

Animals under human care can be studied longitudinally (up to approximately 17 years in this study) and may provide a more accurate baseline calculation by averaging hormone values over the lifetime of the animal. A longitudinal study provides data that may be used as a relative indication of change over time. Understanding differences in baseline FGM levels and between individuals has implications for zoo and aquarium management. For example, biologists can make husbandry decisions catered to individuals that minimize stress responses by understanding the individual variation in FGM response, baseline, and temperament. To assess stress, a combination of FGMs, behavior, and health should be taken into consideration. We found that endocrine profiles of animals at the SA revealed acute peaks of the FGM response to various external stimuli and no evidence of a chronic stress response. 

Sea otters under human care are ambassadors for their counterparts in the wild. Sea otters are keystone species that structure nearshore marine ecosystems. As they reoccupy portions of their historic range, kelp forest communities recover, making for a more productive and stable nearshore environment [48]. Sea otters in the wild will continue to be exposed to various anthropogenic stressors such as vessel traffic, pollution, and oil spills, as well as future effects from climate change. The ability to measure FGMs as a non-invasive measure for acute and chronic stress level may be an important tool for monitoring the overall health of this iconic important species in human care and in the wild.

## 5. Conclusions

This study successfully analytically validated the use of two commercially available EIA kits to measure stress-related fecal cortisol-immunoreactive and corticosterone-immunoreactive metabolites, in northern sea otter fecal samples. Four individual sea otters were studied, and baseline calculations for each fecal glucocorticoid metabolite (FGM) were calculated for each animal. Each individual’s baseline level was found to be unique. Some peaks in FGMs over a longitudinal time period were matched with notes on environmental, husbandry, or veterinary care.

This work is important for understanding which external stimuli may cause stress responses in animals under human care. Monitoring baseline FGM levels may provide insight into the effective husbandry management of sea otters to improve the overall welfare of these marine mammals.

## Figures and Tables

**Table 1:** Samples collected during the study period.

Animal ID	Sex	Age Sampled (Years)	N Fecal Samples
M1	M	9–17 (2008–2016)	233
F1	F	2–19 (1999–2016)	122
F2	F	4–14 (2006–2016)	72
F3	F	1–2 (2015–2016)	60

**Table 2:** Sea otter FGM baseline calculations for fecal cortisol and corticosterone metabolites. Number of peaks for each hormone metabolite is reported, and number of peaks in both. Peaks in FGMs were defined as >2SD above the mean. The number of associated notes to both metabolite peaks and detail are also reported. Older samples and dates did not have many correlating notes and throughout the years, more notes were taken (including normal behavior). Enrichment items may include ice treats or feeder puzzles, sound stimulation, or toys.

Animal ID	Fecal Cortisol- Immunoreactive Metabolite Baseline (ng/g)	Fecal Corticosterone- Immunoreactive Metabolite Baseline (ng/g)	Fecal Cortisol- Immunoreactive Metabolite Peaks	Fecal Corticosterone- Immunoreactive Metabolite Peaks	Both FGM Peaks	Peak Note	Note Detail
M1	38.3	59.7	33	26	11	1	Deslorelin implants and associated vet exam
F1	83.7	102	4	16	0	NA	NA
F2	32.8	54.4	8	13	5	1	Vet exam
F3	20.2	52.3	7	9	7	7	Arrival at SA, vet exam, normal behavior, shifted exhibit (2), and enrichment (2)

## Data Availability

Please contact the corresponding author for the data files.

## Supplementary Materials

The following supporting information can be downloaded at: https://www.mdpi.com/article/10.3390/ani13132175/s1, Table S1: Enzyme immunosorbent assay details of commercially available kits (Enzo Life Sciences Inc., New York, USA). All information is provided in kit inserts by the manufacturer.
