# Supplementary material for: Analytical Validation and Assessment of Baseline Fecal Glucocorticoid Metabolites in Northern Sea Otters (Enhydra lutris kenyoni) in Human Care
Source: Animals (Basel). 2023 Jul 2;13(13):2175. doi: 10.3390/ani13132175 (PMC10339873; doi:10.3390/ani13132175)
Supplement: Supplementary file 1 [file animals-13-02175-s001.zip › animals-2410112-supplementary.pdf]

Supplement

Table S1: Enzyme immunosorbent assay details of commercially available kits (Enzo Life Sciences Inc., New York, USA). All information is provided in kit inserts by the manufacturer.

**Hormone (assay catalog number)**	**Antibody Source**	**Standard Range**	**Sensitivity**	**Intra-Assay %CV**	**Inter-Assay %CV**	**Cross Reactivities**
Cortisol<br>(ADI-900-071)	Mouse monoclonal	100 ng/g<br>50 ng/g<br>25 ng/g<br>12.5 ng/g<br>6.25 ng/g<br>3.13 ng/g<br>1.56 ng/g	0.5672 ng/g	6.6 – 10.5%	7.8 – 13.4%	Cortisol 100%<br>Prednisolone 122.35%<br>Corticosterone 27.68%<br>11-deoxycortisol 4.0%<br>Progesterone 3.64%<br>Prednisone 0.85%<br>Testosterone 0.12%<br>Androstenedione < 0.1%<br>Cortisone < 0.1%<br>Estradiol < 0.1%
Corticosterone<br>(ADI-900-097)	Sheep polyclonal	200 ng/g<br>40 ng/g<br>8 ng/g<br>1.6 ng/g<br>0.32 ng/g	0.2699 ng/g	6.6 – 8.4%	7.8 – 13.1%	Corticosterone 100%<br>Deoxycorticosterone 28.6%<br>Progesterone 1.7%<br>Testosterone 0.13%<br>Tetrahydrocorticosterone 0.28%<br>Aldosterone 0.18%<br>Cortisol 0.046%<br>Pregnenolone <0.03%<br>β-Estradiol <0.03%<br>Cortisone <0.03%<br>11-dehydrocorticosterone acetate <0.03%
